# Characteristics of antimicrobial peptide OaBac5mini and its bactericidal mechanism against *Escherichia coli*

**DOI:** 10.3389/fvets.2023.1123054

**Published:** 2023-02-23

**Authors:** Shanshan Shen, Yawei Sun, Fei Ren, Jessica M. A. Blair, Pauline Siasat, Shuaiqi Fan, Jianhe Hu, Junping He

**Affiliations:** ^1^College of Veterinary Medicine, Shanxi Agricultural University, Taigu, Shanxi, China; ^2^College of Animal Science and Veterinary Medicine, Henan Institute of Science and Technology, Xinxiang, Henan, China; ^3^Institute of Microbiology and Infection, College of Medical and Dental Sciences, University of Birmingham, Birmingham, United Kingdom

**Keywords:** antimicrobial peptide OaBac5mini, biochemical characteristics, physical characteristics, *Escherichia coli*, bactericidal mechanism

## Abstract

**Introduction:**

Antimicrobial peptides (AMPs) play an important role in defending against the attack of pathogenic microorganisms. Among them, the proline-rich antibacterial peptides (PrAMPs) have been attracting close attention due to their simple structure, strong antibacterial activity, and low cell toxicity. OaBac5mini is an active fragment of the sheep-derived OaBac5 belonging to the PrAMPs family.

**Methods:**

In this study, the antibacterial activity of OaBac5mini was investigated by testing the MICs against different stains of *E. coli* and *S. aureus* as well as the time-kill curve. The bactericidal mechanism was explored by determining the effect of OaBac5mini on the cell membrane. The stability and biosafety were also evaluated.

**Results:**

The susceptibility test demonstrated that OaBac5mini showed potent antibacterial activity against the multidrug-resistant (MDR) *E. coli* isolates. It is noticeable that the absence of inner membrane protein SbmA in *E. coli* ATCC 25922 caused the MIC of OaBac5mini to increase 4-fold, implying OaBac5mini can enter into the cytoplasm *via* SbmA and plays its antibacterial activity. Moreover, the antibacterial activity of OaBac5mini against *E. coli* ATCC 25922 was not remarkably affected by the serum salts except for CaCl_2_ at a physiological concentration, pH, temperature, repeated freeze-thawing and proteases (trypsin < 20 μg/mL, pepsin or proteinase K). Time-kill curve analysis showed OaBac5mini at the concentration of 200 μg/mL (8 × MICs) could effectively kill *E. coli* ATCC 25922 after co-incubation for 12 h. In addition, OaBac5mini was not hemolytic against rabbit red blood cells and also was not cytotoxic to porcine small intestinal epithelial cells (IPEC-J2). Bioinformatic analysis indicated that OaBac5mini is a linear peptide with 8 net positive charges. Furthermore, OaBac5mini significantly increased the outer membrane permeability and impaired the inner membrane integrity and ultrastructure of *E. coli* ATCC25922.

**Conclusion:**

OaBac5mini is a stable and potent PrAMP that kills *E. coli* by two different modes of action - inhibiting intracellular target(s) and damaging cell membrane.

## Introduction

*Escherichia coli* (*E. coli*) is a rod-shaped Gram-negative bacterium belonging to Enterobacteriales, Enterobacteriaceae, *Escherichia*, which includes commensal and pathogenic strains. *Escherichia coli* can cause a variety of diseases in humans and animals, such as diarrhea, extraintestinal diseases (1), urinary tract infection (UTI) and bovine mastitis (2, 3). However, with the emergence and spread of resistant *E. coli*, treatment options are becoming more limited (4). Therefore, finding alternatives to antibiotics to treat pathogenic *E. coli* is becoming more important for both humans and animals.

Antimicrobial peptides (AMPs) are small molecular peptides in living organisms and serve as natural barriers against the invasion of pathogenic microorganisms (5). They are often referred to as cationic host defense peptides (HDPs) or cationic antimicrobial peptides (CAMPs) (6). Cathelicidins are one of the best characterized AMP families. Each cathelicidin includes an N-terminal signal domain, a cathelin-like domain, and an antimicrobial C-terminal domain (7). Among them, the proline-rich antibacterial peptides (PrAMPs) have been attracting the close attention of researchers due to their strong antibacterial activity against Gram-negative bacteria, remarkably low toxicity toward mammalian cells, and the lack of extensive membrane-damaging effects (8). Bac5 is the first member of the PrAMPs to be identified in cattle, sheep, and goats (9, 10). Moreover, its homologous peptides have been subsequently found in another mammal (deer) (11). It has been demonstrated that mature Bac5 is a linear peptide composed of 43 amino acid residues (12) which includes a critical element for antibacterial activity containing 15 Arg-rich residues (13). In 2018, Mardirossian et al. (10) confirmed that Bac5 (1–25) and Bac5 (1–31), two active fragments of Bac5, inhibited bacterial protein synthesis by binding to ribosome after entertaining into the cytoplasm of *E. coli* BW25113 *via* an inner membrane transporter SbmA. In addition, OaBac5, a homologous PrAMP of Bac5 was first isolated from the crude neutrophil extraction from ovine blood in 2003. Based on its cDNA sequence, OaBac5 is a 51-residue PrAMP (14). Research showed that OaBac5 exhibited potent activity against *E. coli* 0157:H7, *Staphylococcus aureus* 1056 MRSA, and *Candida albicans* 3153A (MICs, 4–10 μg/ml) (15). Likewise, two other variants (OaBac5α and OaBac5β) of OaBac5 were isolated from elastase-treated extracts of sheep leukocytes which showed potent activity against bacteria and fungi (16). In 2004, OaBac5mini was first synthesized as a truncated fragment of OaBac5, comprising the first 24 N-terminal residues. OaBac5mini exhibited potent activity against Gram-negative bacteria (MICs, 0.125–8 μg/ml) but weak activity against Gram-positive bacteria and *C. albicans* (MICs, 16–64 μg/ml), which was similar to that of OaBac5. However, compared to Bac5, OaBac5mini was more active against Gram-negative bacteria (17). In addition, the antibacterial activity of OaBac5mini was not significantly affected by pH or temperature. Moreover, it also showed synergistic activity with other PrAMPs (SMAP29 and OaBac7.5mini), polymyxin B, and lysozyme against *E. coli* 0157:H7 and *S. aureus* 1056 MRSA (18, 19). The above-mentioned results imply OaBac5mini has the potential to become a promising lead compound for new antibiotic substitutes. However, the characteristics and bactericidal mechanism of OaBac5mini have been unknown and are the subject of this study.

In the present study, we demonstrated that OaBac5mini showed stronger antibacterial activity against MDR *E. coli* isolates than susceptible *E. coli* ATCC 25922. Its antibacterial activity was not significantly affected by *in vitro* conditions factors (pH, temperature, repeated freeze-thawing) and *in vivo* conditions factors (serum salts, pepsin and proteinase K). Moreover, the physicochemical characteristics, the cytotoxicity and the bactericidal mechanism of OaBac5mini were fully investigated in the present study to illustrate the bactericidal action and the safety.

## Materials and methods

### Antimicrobial peptides and antibiotics

Antimicrobial Peptides OaBac5mini (N-RFRPPIRRPPIRPPFRPPFRPPVR-C) was prepared *via* solid-phase synthesis using 9-fluorenylmethoxycarbonyl (F-moc) chemistry at GL Biochem (Shanghai) Ltd. and analyzed by HPLC and MALDI-TOF MS to confirm that the purity was >91.06%.

Amoxicillin, kanamycin, florfenicol, and tetracycline were purchased from China National Institute for Drug and Biological Products Control (Beijing, China). Polymyxin B sulfate (PMB) purchased from Beijing Solarbio Science & Technology co., Ltd (Beijing, China).

### Bacterial strains and cells

The strains of *E. coli* ATCC 25922 and *S. aureus* ATCC 25923 were purchased from the American Type Culture Collection (ATCC, Manassas, VA, United States). The strains of *E. coli* W13, *E. coli* IF4, *E. coli* 2, and *E. coli* 17 were individually isolated from the livers of diseased chickens infected by *E. coli*. The strains of *S. aureus* B5, *S. aureus* B9, *S. aureus* B54, and *S. aureus* B63 were individually isolated from the milk of cows with mastitis. These clinical isolates were all identified by the VITEK-32 system (bioMérieux, France) and PCR amplification sequencing.

IPEC-J2 cells were obtained from American Type Culture Collection (ATCC, Manassas, VA, United States). IPEC-J2 cells were cultured in High Glucose DMEM (Dulbecco's Modified Eagle Medium, HyClone, USA) supplemented with 10% fetal bovine serum (Biological Industries, Israel), and 1% penicillin-streptomycin (Solarbio, China). The cells were maintained in CO_2_ incubator (Galaxy R, RS Biotech, Scotland) with 5% CO_2_ at 37°C.

### Construction of *E. coli* ATCC 25922 Δ*sbmA*

The method described by Datsenko and Wanner was used for chromosomal gene deletion (20). In brief, the genetic fragment containing the kanamycin resistance gene *aph* flanked by FLP Recognition Target (FRT) sites was amplified by PCR using the template plasmid pKD4 and the hybrid primers LBMAF:5′-GGCAG ATCCCGATTAGCGCCGCGCGTTTCTGGTCGTTGGATTTCCGTGTAGGCTGGAGCTGCTTC-3′ and LBMAR: 5′-CTCGCGTACCGTAGGCGGCGTCGCGCGCGTGGCATCGTCTTCACCCATATGAATATCCTCCTTAG-3′, which consisted of 20 nucleotides (nt) of the helper plasmid pKD4 and 45 nt on the 5′ and 3′ ends of the inactivated *sbmA*. The PCR fragment (1,567 bp) was purified, digested with DpnI, repurified and transferred into *E. coli* ATCC 25922 by electroporation, in which the Red recombinase expression plasmid pKD46 was previously transformed and induced by L-arabinose. Transformants were selected on Luria-Bertani (LB) agar containing 50 μg/ml of kanamycin at 37°C. The inserted sequence was amplified from the kanamycin-resistant strains by using the primers SBMF: 5′-GCACGGCAGAAAAAAGCA-3′ and SBMR: 5′-GACGGAAACAGCAAGAACAAA-3′ which is located outside of inactivated gene. The length of the PCR product of *sbmA* using the primers set SBMF and SBMR in *E. coli* ATCC 25922 is 1,404 bp. When *sbmA* is successfully inactivated, the PCR product (2,198 bp) was amplified and sequenced for the verification of the gene deletion.

### Antibacterial activity

MICs of OaBac5mini, amoxicillin, kanamycin, florfenicol and tetracycline were determined using the two-fold broth microdilution method according to the Clinical and Laboratory Standards Institute (CLSI) guidance (21). MIC values of the tested antibacterial agents were determined on three independent occasions. *Escherichia coli* ATCC 25922 was used as the quality control in all the susceptibility tests.

### Salt sensitivity assay

To determine the effect of different salt ions on the antibacterial activity of OaBac5mini, MIC values of OaBac5mini were tested at different physiological salt concentrations (150 mM NaCl, 4.5 mM KCl, 6 μM NH_4_Cl, 1 mM MgCl_2_, 2.5 mM CaCl_2_, and 4 μM FeCl_3_) as described by Maisetta et al. (22).

### *In vitro* and *in vivo* conditions factors sensitivity assay

The sensitivities of OaBac5mini to different pH, temperature, digestive enzymes, and repeated freeze-thawing conditions were determined using the inhibition zone assay as previously described (23). For pH sensitivity, OaBac5mini was diluted with each solution whose pH was individually adjusted to 2, 3, 4, 5, 6, 7, 8, 9, 10, 11 with HCl or NaOH, and incubated for 2 h, followed by adjusting the pH to 7 to end the reaction. For temperature sensitivity, the diluted peptide was incubated at 30, 40, 50, 60, 70, 80, 90, and 100°C for 30 min. To determine the digestive enzymes sensitivity, OaBac5mini was incubated with 100 μg/ml pepsin (in pH 2 sterile water, sigma, USA), 5–100 μg/ml trypsin (in pH 6.8 KH_2_PO_4_ buffer, sigma, USA) and 100 μg/ml protease K (Solarbio, China) at 37°C for 3 h, respectively. To end the reaction after incubation, the pepsin buffer was adjusted to pH 8, the trypsin solution and protease K solution were incubated at 95°C for 10 min. For repeated freeze-thawing sensitivity, OaBac5mini was frozen and then thawed at room temperature 0–12 times. All the final concentrations of OaBac5mini in sensitivity tests were 50 μg/ml. All experiments were repeated three times.

### Time-kill curve

To investigate the time-kill effect of OaBac5mini against *E. coli* ATCC 25922, bacteria (5×10^5^ CFU/ml) were cultured in fresh Mueller-Hinton (MH) broth (Yashi Organisms, China) with different concentrations of OaBac5mini (0, 50, 100, and 200 μg/ml) at 37°C with shaking at the speed of 220 rpm. Equal amounts (100 μl) of *E. coli* ATCC 25922 from each sample were withdrawn at 0, 2, 4, 6, 8, 10, 12, and 24 h, diluted with Luria-Bertani (LB) broth (Solarbio, China) and plated onto LB agar plates. Plates were placed at 37°C for 18 h for the CFU counts.

### Hemolysis assay

The fresh blood was collected from rabbit hearts, and then centrifuged and rinsed with phosphate-buffered saline (PBS) three times. The 1:100 diluted rabbit blood cells (RBCs) solution was mixed with an equal volume of OaBac5mini solution (3.125–400 μg/ml final concentrations), PBS (negative control) and 0.1% Triton X-100 (Solarbio, China, positive control), respectively, followed by incubating at 37°C for 1 h. The mixed solution was centrifuged and 100 μl of the supernatant was added into a 96-well plate. The optical density (OD) of samples was monitored immediately using INFINITE 200 PRO (Infinite^®^ 2000, TECAN, Austria) at an absorption wavelength of 413 nm. The experiment was conducted in triplicate. The OD value at 413 nm was used to calculate the hemolysis ratio with the formula below:


$$\begin{array}{l} Hemolysis\;ratio=\left(OD_{AS}-OD_{AN}\right)÷\left(OD_{AP}-OD_{AN}\right)\times 1\% \end{array}$$

AS is the OaBac5mini-treated sample, AN is the negative control, AP is the positive control.

### Real-time cell assay

Real time cellular analysis (RTCA) was used to evaluate the proliferation and viability of IPEC-J2 cells. Cells were seeded in 16-well E-plates (xCELLigence, ACEA biosciences, USA) at the concentration of 20,000 cells per well, allowing attachment overnight, and then high glucose MEM was replaced by fresh complete MEM with different concentrations of OaBac5mini (25, 50, 100, 200, and 400 μg/ml). Real time cellular analysis was monitored using xCELLigence RTCA DP system (xCELLigence, ACEA biosciences, USA) for 100 h at 15 min intervals. The cell index was normalized by RTCA software 2.0 (xCELLigence, ACEA biosciences, USA).

### Physicochemical properties analysis and structure prediction

To investigate the antibacterial mechanism of OaBac5mini, the physicochemical properties of OaBac5mini were analyzed using ProtParam software (https://www.expasy.org/resources/protparam/) in protein and proteomes resources of ExPASy (24), the Swiss Bioinformatics Resource Portal. Furthermore, the hydrophilic and hydrophobic regions were predicted using the ProtScale tool with Hphob./Kyte & Doolittle scale (https://web.expasy.org/protscale/). The secondary structure was predicted by PHD on the NPS@ server (https://npsa-prabi.ibcp.fr/NPSA/npsa_phd.html). The three-dimension (3D) model was predicted by PEP-FOLD3 (https://bioserv.rpbs.univ-paris-diderot.fr/services/PEP-FOLD3/).

### Permeability of the outer membrane

The permeability of OaBac5mini to *E. coli* ATCC 25922 cell membranes was determined by N-Phenyl-1-naphthylamine (NPN, sigma, India), which is a fluorescent dye that only can pass through the damaged cell membrane and exhibit an increase of fluorescence. The NPN uptake assay was conducted following the instructions of Helander and Mattila (25) with minor modifications. In brief, *E. coli*. ATCC 25922 in logarithm growth period was centrifuged and resuspended with N′-a-hydroxythylpiperazine-N′-ethanesulfanic acid (HEPES, Solarbio, China, 5 mM, pH 7.2–7.4). NPN (10 μM final concentration, diluted with 5 mM HEPES) containing OaBac5mini (25, 50, 100, and 200 μg/ml final concentrations), PMB (positive control, 50 μg/ml final concentrations) or PBS (negative control) were mixed with an equal volume of prepared bacteria solution and were added into a 96-well plate. Fluorescence units were monitored immediately using INFINITE 200 PRO (Infinite^®^ 2000, TECAN, Austria) at the excitation wavelength of 350 nm and emission wavelength of 420 nm for 100 min at 5 min intervals. Experiments were performed in biological triplicate.

### Integrity of the inner membrane of cell

The integrity of the inner membrane of *E. coli* ATCC 25922 treated by OaBac5mini was investigated by 3,3′-Dipropylthiadicarbocyanine iodide [DiSC_3_(5), Sigma, USA], which is a fluorescent probe that can be concentrated in the integrated cytoplasmic membrane, leading to fluorescence self-quenching. If the membrane is depolarized or disrupted, DiSC_3_(5) is released into the aqueous medium and leads to increased fluorescence. The DiSC_3_(5) assay was conducted following the instruction of Sautrey et al. (26) with minor modifications. In brief, *E. coli* ATCC 25922 was centrifuged and diluted with 5 mM HEPES (containing 5 mM glucose) till OD_600_ is 0.05. 1 μM DiSC_3_(5) was added into the prepared bacteria solution, then incubated at 37°C with shaking at 220 rpm for 1 h in the dark. KCl solution was added (final concentration was 100 mM) after 30 min incubation. Then, 190 μl treated bacteria solution and either 10 μl OaBac5mini (the final concentrations were 25, 50, 100, and 200 μg/ml), PMB (positive control, final concentrations were 50 and 100 μg/ml), or PBS (negative control) were added into wells in a 96-well plate. Fluorescence units were investigated immediately using INFINITE 200 PRO (Infinite^®^ 2000, TECAN, Austria) at the excitation wavelength of 620 nm and emission wavelength of 670 nm for 30 min at 1 min intervals. Triple experiments were conducted with three individual clones of *E. coli* ATCC 25922.

### Scanning electron microscopy

*Escherichia coli* ATCC 25922 (OD_600_ = 0.6) was centrifugated and resuspended with the same volume of 10 mM PBS (negative control), OaBac5mini (50 and 200 μg/ml) and PMB (50 μg/ml, positive control), respectively, and then incubated at 37°C for 3 h. The treated bacteria were collected by centrifugation and rinsed with PBS (PH 7.4) three times, then fixed with 2.5% glutaraldehyde overnight at 4°C. The fixed bacteria were centrifugated and dehydrated for 15 min with a series of gradient ethanol solutions (30, 50, 70, 80, 90, 95, and 100%), and then resuspended with 100 μl 100% ethanol. Three microliter of resuspended bacteria were dripped onto the treated silicon wafer and the bacteria were observed using scanning electron microscopy (SEM; Quanta 200, FEI, USA).

### Statistical analysis

The statistical analyses were performed using GraphPad Prism 5.0 (GraphPad Software, San Diego, CA) and shown with MEAN ± SEM. One-way ANOVA was used to test the statistical significance of the differences, and then Dunnett's multiple comparison test or Tukey's multiple comparison test was performed using SPSS 20 (SPSS, Chicago, USA). Statistically significance defined as ^*^P < 0.05, ^**^P < 0.01, and ^***^P < 0.001.

## Results

### Antibacterial activity of OaBac5mini

The MICs of OaBac5mini, kanamycin, amoxicillin, florfenicol and tetracycline against different strains were shown in Table 1. OaBac5mini showed potent antibacterial activity against *E. coli* (MICs, 1.95–25 μg/ml) but weak antibacterial activity against *S. aureus* (MICs, 208.33 to >250 μg/ml). Moreover, OaBac5mini exhibited stronger activity against clinical isolates of MDR *E. coli* (MICs, 1.95–3.26 μg/ml) than *E. coli* ATCC 25922 (MIC, 25 μg/ml). To be noticed, the absence of *sbmA* caused the MIC of *E. coli* ATCC 25922 to OaBac5mini increase four-fold, suggesting that OaBac5mini can enter into the cytoplasm *via* SbmA and plays its antibacterial activity.

**Table 1:** The MICs of OaBac5mini and antibiotics against strains (unit: μg/ml).

**Strains**	**Source**	**Antibacterial agents**				
		**OaBac5mini**	**KAN**	**AMO**	**FLO**	**TET**
*E. coli* ATCC 25922	American Type Culture Collection	25	4	8	4	2
*E. coli* ATCC 25922 Δ*sbmA*	This study	100	ND	8	5.3	1.67
*S. aureus* ATCC 25923	American Type Culture Collection	>250	4	0.67	16	1
*E. coli* W13	Diseased chicken liver	3.26	320	>640	320	160
*E. coli* IF4	Diseased chicken liver	2.60	>640	>640	80	133.33
*E. coli* 2	Diseased chicken liver	1.95	>640	>640	266.67	1
*E. coli* 17	Diseased chicken liver	3.26	320	>640	80	160
*S. aureus* B5	The milk of cows with mastitis	>250	40	426.67	4.17	0.08
*S. aureus* B9	The milk of cows with mastitis	250	186.67	3.33	1.67	80
*S. aureus* B54	The milk of cows with mastitis	250	5.83	3.33	2.5	66.67
*S. aureus* B63	The milk of cows with mastitis	208.33	1.25	2.08	1.04	0.42

AMO, amoxicillin; KAN, kanamycin; FLO, florfenicol; TET, tetracycline; ND, not determined.

The antibacterial activity of OaBac5mini against *E. coli* ATCC 25922 was investigated in the presence of different salt ions with similar concentrations to that in human serum. As shown in Table 2, the presence of increased CaCl_2_ decreased susceptibility to OaBac5mini compared to MH broth alone. Other salt ions (MgCl_2_, NH_4_Cl, FeCl_3_, NaCl and KCl) did not significantly affect the antibacterial activity of OaBac5mini. This suggests that OaBac5mini will show a comparable stability in serum.

**Table 2:** MICs of OaBac5mini in the presence and absence of physiological concentrations of different serum salts (unit: μg/ml).

**Culture medium**	***E. coli* ATCC 25922**
MH broth + NaCl (150 mM)	33.33
MH broth + KCl (4.5 mM)	20.83
MH broth + NH_4_Cl (6 μM)	12.5
MH broth + MgCl_2_ (1 mM)	50
MH broth + CaCl_2_ (2.5 mM)	100
MH broth + FeCl_3_ (4 μM)	16.67
MH broth	25

**Figure 1:**
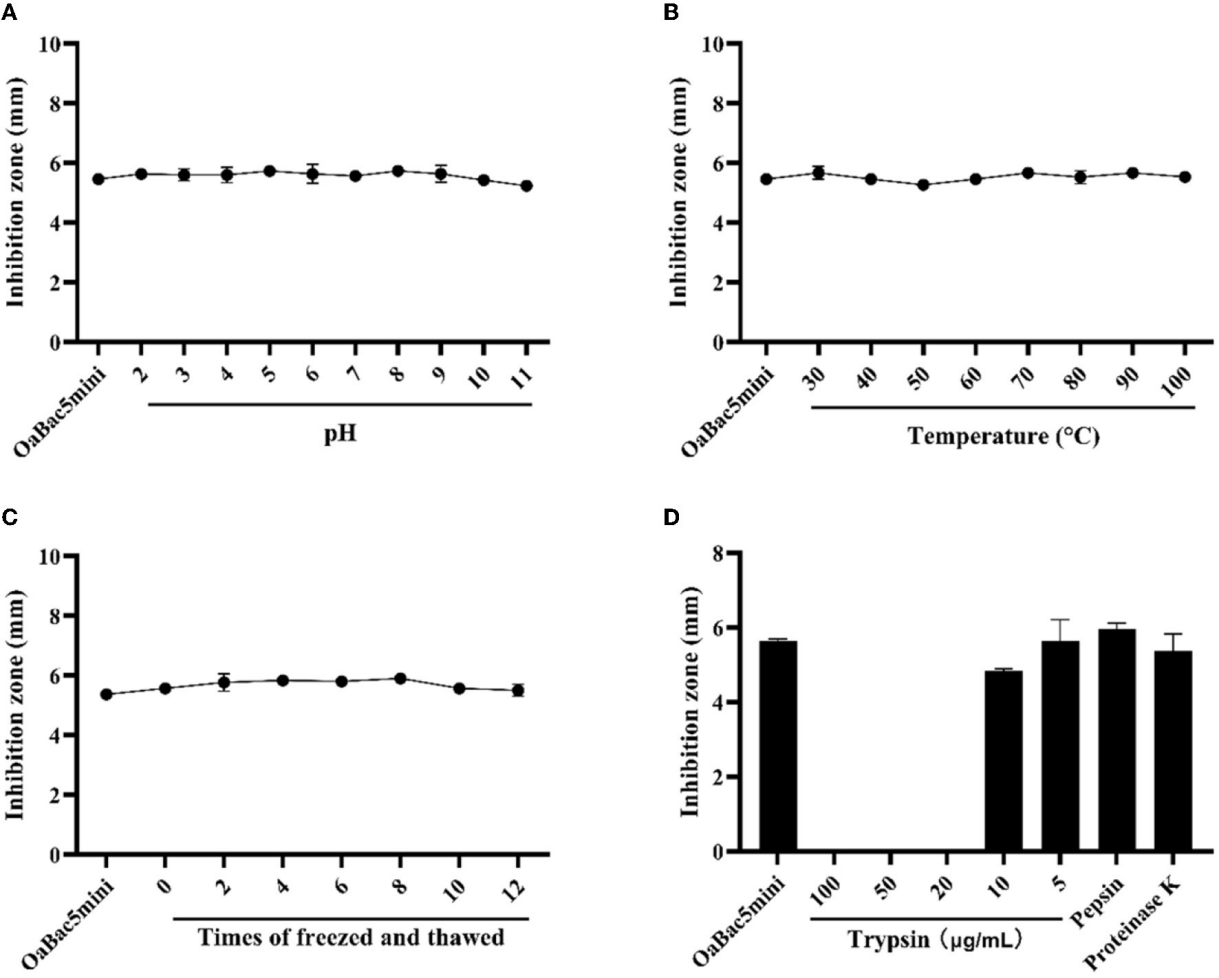
The sensitivities of *E. coli* ATCC 25922 to antimicrobial peptide OaBac5mini at different conditions. **(A)** The effect of solution pH on the MIC of OaBac5mini. **(B)** The effect of temperature on the MIC of OaBac5mini. **(C)** The effect of repeated freeze-thawing on the MIC of OaBac5mini. **(D)** The effect of trypsin, pepsin and proteinase K on the MIC of OaBac5mini. Error bars represent means ± SEM. Assays were performed in triplicate.

As shown in Figure 1, OaBac5mini exhibits stable antibacterial activity against *E. coli* ATCC 25922 in acid-base environments (Figure 1A), high-temperature environments (Figure 1B), repeated freeze-thawing (Figure 1C) or digestive enzymes pepsin and proteinase K as well as trypsin with low concentrations (Figure 1D). However, OaBac5mini completely lost activity after it was treated by trypsin with concentrations ranging from 20 to 100 μg/ml.

### Time-kill curve of OaBac5mini

**Figure 2:**
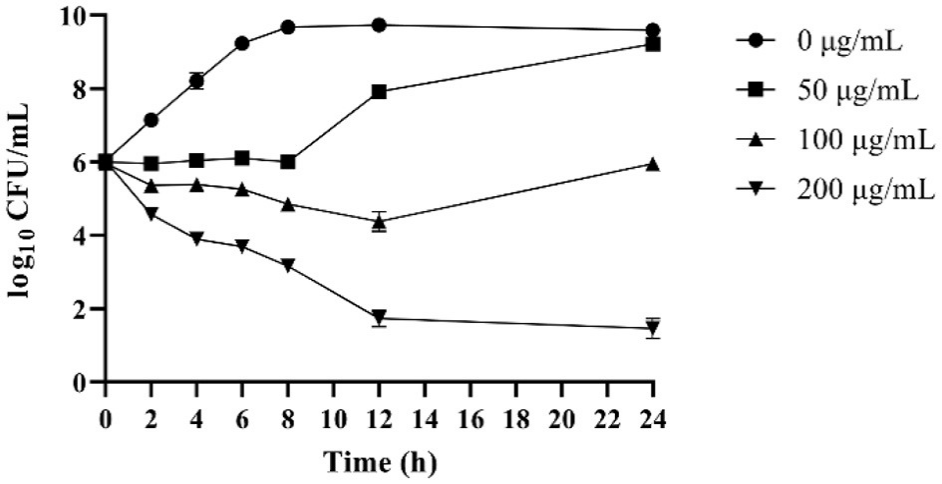
Time-dependent killing curves of *E. coli* ATCC 25922 in the presence of different concentrations of OaBac5mini (0, 50, 100, and 200 μg/ml) in MH broth medium for 24 h. Aliquots were collected at 0, 2, 4, 6, 8, 10, 12, and 24 h to count the bacteria. Error bars represent means ± SEM. Assays were performed in triplicate.

The time-kill curves of OaBac5mini against *E. coli* ATCC 25922 are shown in Figure 2. Results showed that 200 μg/ml OaBac5mini (8 × MIC) effectively killed *E. coli* ATCC 25922 after co-incubation for 12 h. When the concentration of OaBac5mini was 50 μg/ml (2 × MIC), the number of bacteria remained stable in the first co-incubation for 8h before it increased, attaining an amount similar to that of *E. coli* ATCC 25922 without OaBac5mini after co-incubation for 24 h. At the same time, 100 μg/ml OaBac5mini also caused the numbers of bacteria to decrease after co-incubation for 12 h and increase in further co-incubation for 12 h.

### Hemolysis and cytotoxicity of OaBac5mini

**Figure 3:**
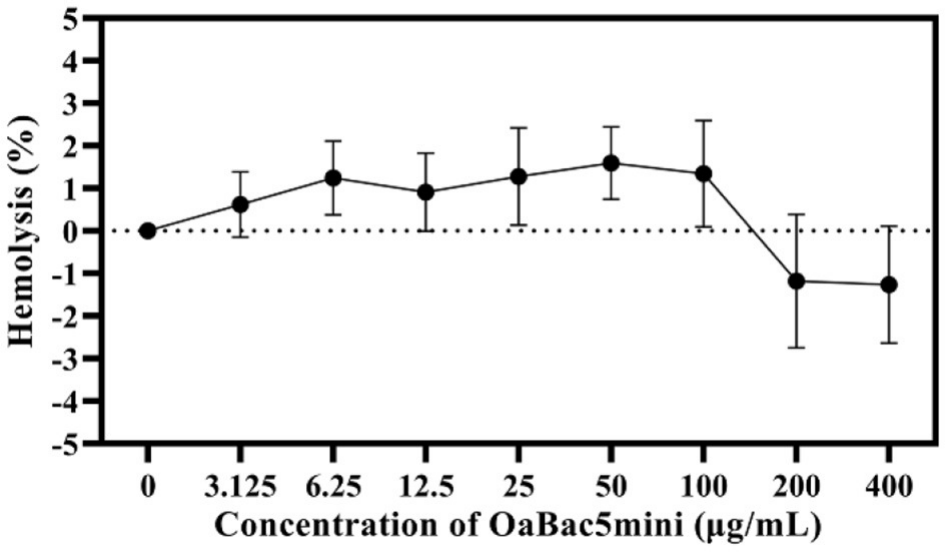
The hemolysis rate of antimicrobial peptide OaBac5mini. All data were measured at the maximum absorption wavelength (415 nm) at least three times. Error bars represent means ± SEM. There was no significant difference between OaBac5mini treated groups and the negative control group (*P* > 0.05).

The hemolysis of OaBac5mini against RBCs was decided by testing the OD of every sample at an absorption wavelength of 413 nm. As shown in Figure 3, the mean hemolysis rate of RBCs was −1.26 to 1.59% after co-incubating with different concentrations of OaBac5mini (3.125–400 μg/ml) at 37°C for 1 h, which indicates that OaBac5mini does not cause hemolysis.

**Figure 4:**
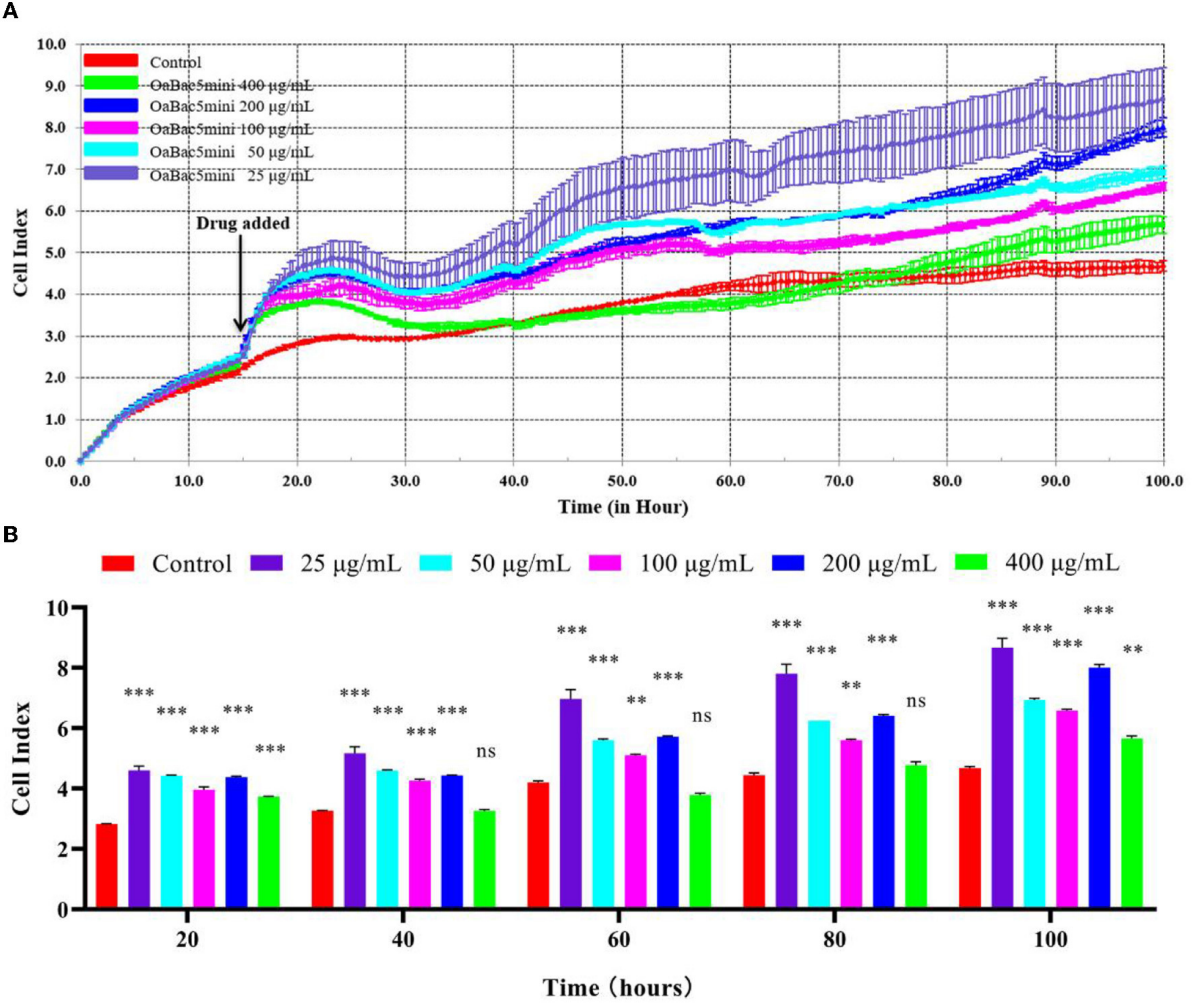
The toxicology of OaBac5mini on IPEC-J2 cells. **(A)** The cell index of IPEC—J2 cells. The black arrow indicates the time point of adding the peptides. The cells without antimicrobial peptide were used as the negative control. **(B)** Parallel comparisons of the survival rates of IPEC-J2 cells treated by OaBac5mini with 25, 50, 100, 200, and 400 μg/ml at five specific time points. Error bars represent means ± SEM, *n* = 3. ** and *** represent very significant and extremely significant, respectively (*P* < 0.01 and *P* < 0.001 by Two-way ANOVA; ns represents no significance).

The results of RTCA showed that OaBac5mini was not cytotoxic to IPEC-J2 cells, but seemed to increase the proliferation of cells (Figure 4). Overall, the cell indexes of OaBac5mini treated groups showed a significant increase (*P* < 0.01) compared with the control group (Figure 4B). The lower the concentration of OaBac5mini, the stronger the promotion effect on cell proliferation was observed (Figure 4A). Even though the concentration of OaBac5mini attained 400 μg/ml, the cell indexes didn't change significantly compared to the control group (*P* > 0.05; Figure 4B).

### Physicochemical properties analysis and structure prediction

**Figure 5:**
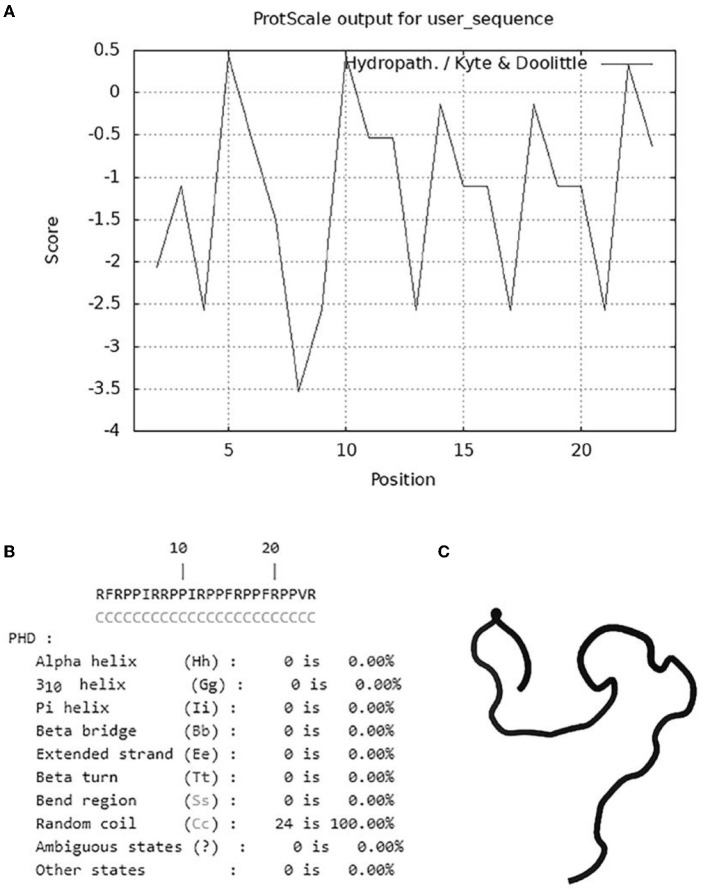
The hydrophilic and hydrophobic properties analysis and structure prediction. **(A)** The hydrophobicity of OaBac5mini was analyzed by ProScale. Scores below zero represent hydrophilicity. **(B)** The secondary structure of OaBac5mini predicted by PHD. Letter C represents random coil. **(C)** The 3D structure of OaBac5mini predicted by PEP-FOLD3 showed a linear structure without α-helix or β-sheet.

To illustrate the antimicrobial mechanism of OaBac5mini, the physicochemical properties and structure were predicted. As shown in Table 3 and Figure 5, OaBac5mini is a positively charged hydrophilic linear peptide, whose formula is C_142_H_226_N_48_O_25_ with a molecular weight (MW) of 3005.66, as well as a theoretical pI of 12.85. OaBac5mini is a Pro- and Arg-rich peptide, whose amino acid composition is 10 Pro (41.7%), 8 Arg (33.3%), 3 Phe (12.5%), 2 Ile (8.3%), and 1 Val (4.2%). There are 8 positively charged residues, bringing the net charge to 8. Furthermore, the grand average of hydropathicity (GRAVY) is −1.267, and the hydropath./Kyte & Doolittle score predicted by ProtScale was 0.433 to −3.533, which indicates that OaBac5mini is a hydrophilic peptide with several hydrophobic residues (Table 3 and Figure 5A). The predicted secondary structure showed that OaBac5mini is rich in random coils without other secondary structures (Figure 5B). The 3D modeling indicated that OaBac5mini is a linear antimicrobial peptide (Figure 5C).

**Table 3:** The key physicochemical properties of OaBac5mini.

**Parameters**		**Values**
Number of amino acids		24
Formula		C_142_H_226_N_48_O_25_
Molecular weight		3,005.66
	Arg	8 (33.3%)
	Phe	3 (12.5%)
Amino acid composition	Pro	10 (41.7%)
	Ile	2 (8.3%)
	Val	1 (4.2%)
	Others	0%
Theoretical pI		12.85
Grand average of hydropathicity (GRAVY)		−1.267
Total number of negatively charged residues		0
Total number of positively charged residues		8

### Effect of OaBac5mini on the cell membrane

**Figure 6:**
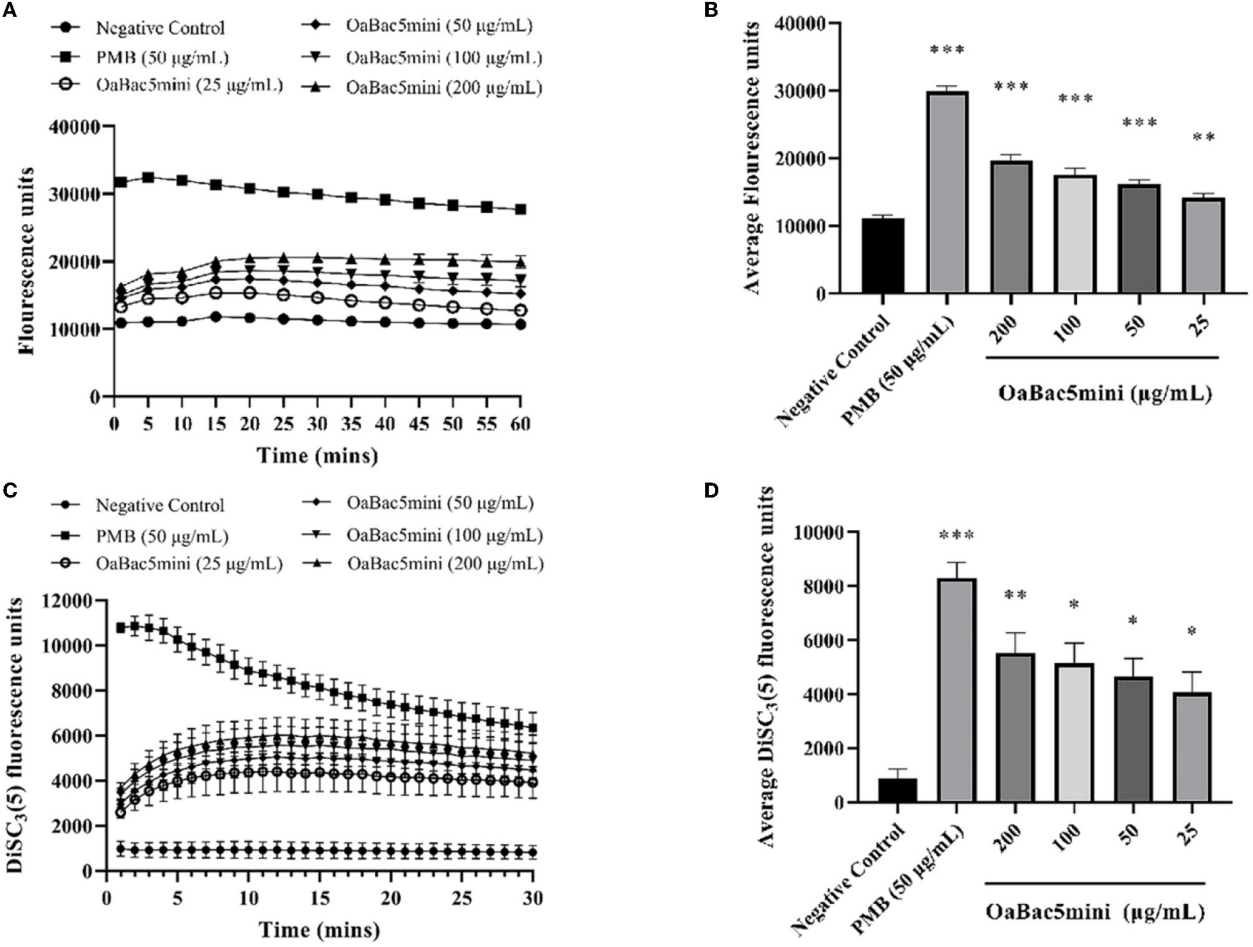
Effects of different concentrations (25, 50, 100, and 200 μg/ml) of OaBac5mini and PMB (50 μg/ml) on the outer membrane permeabilization and inner membrane integrity of *E. coli* ATCC 25922. **(A)** Time–response curve of the outer membrane permeation of *E. coli* ATCC 25922 cells to NPN. **(B)** Comparison of the average NPN fluorescence units (60 min) of *E. coli* ATCC 25922 treated by OaBac5mini with 25, 50, 100, and 200 μg/ml. **(C)** Time–response curve of the inner membrane integrity of *E. coli* ATCC 25922 cells to DiSC_3_(5). **(D)** Comparison of the average DiSC_3_(5) fluorescence units (30 min) of *E. coli* ATCC 25922 treated by OaBac5mini with 25, 50, 100, and 200 μg/ml. Error bars represent means ± SEM, *n* = 3. *, **, and *** represent significant, very significant and extremely significant, respectively (*P* < 0.05, *P* < 0.01, and *P* < 0.001 by One-way ANOVA).

The permeabilization of the outer membrane of *E. coli* ATCC 25922 was determined by detecting the fluorescence changes of NPN. As shown in Figure 6A, the fluorescence intensity increased in bacteria treated with OaBac5mini for 20 min and exhibited a slight decrease until 60 min. The average fluorescence intensity of NPN in bacteria treated with OaBac5mini for 60 min was significantly increased (*P* < 0.01) compared with the negative control (Figure 6B), which indicated that the outer membrane of the bacteria was damaged by OaBac5mini.

The inner membrane integrity of *E. coli* ATCC 25922 was investigated by detecting the fluorescence changes of DiSC_3_(5). As shown in Figure 6C, the fluorescence intensity increased when bacteria were treated with OaBac5mini for 10 min, and gradually leveled off for the next 20 min. The average fluorescence intensity of DiSC_3_(5) of all OaBac5mini treated groups was significantly increased within 30 min (*P* < 0.05, Figure 6D), which indicates that the inner membranes were damaged by OaBac5mini.

**Figure 7:**
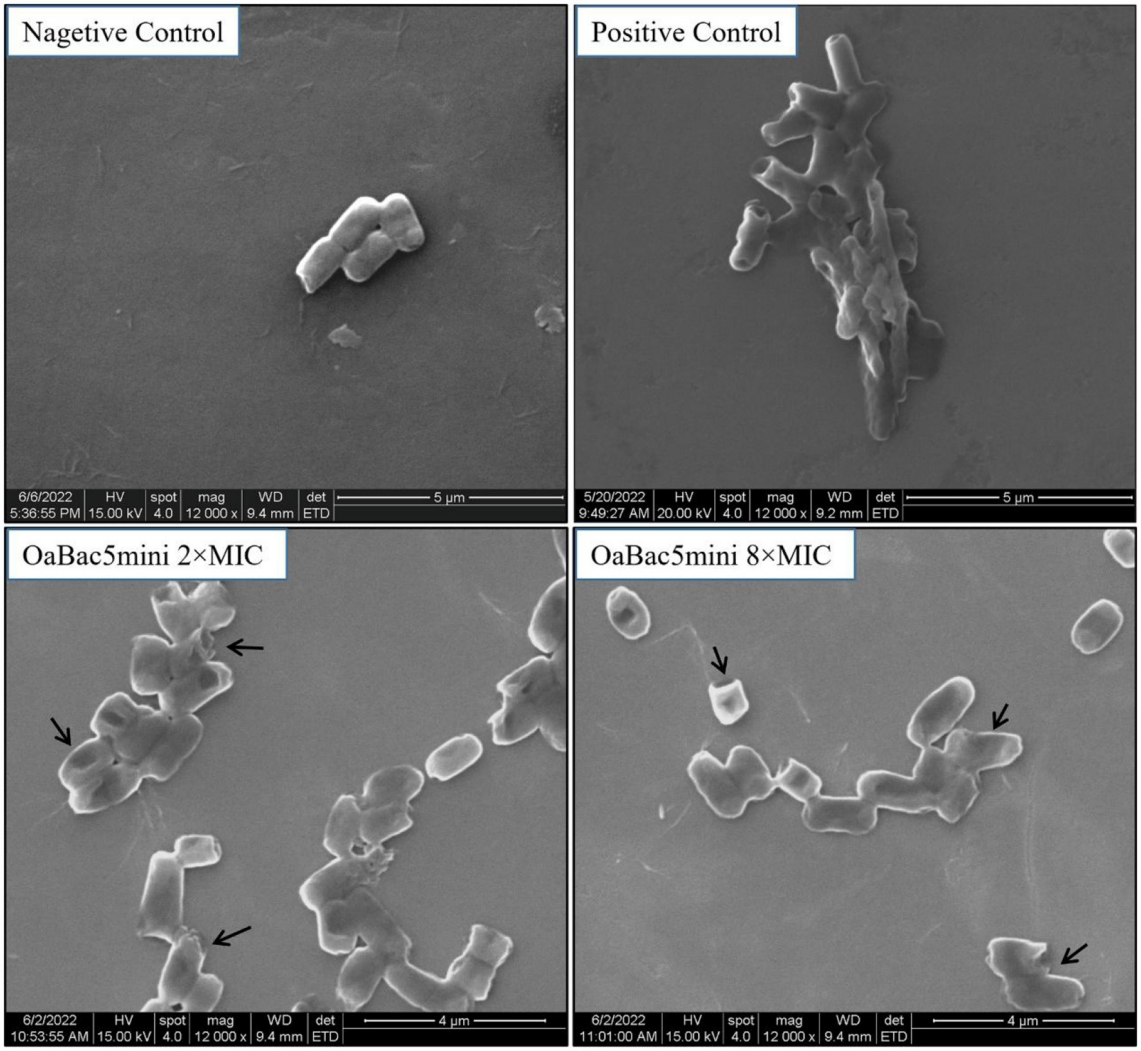
Scanning electron microscopy images of *E. coli* ATCC 25922 treated with OaBac5mini and PMB for 3 h. The bacteria treated with 10 mM PBS is considered as negative control; the bacteria treated with 50 μg/ml PMB is considered as positive control; the bacteria were treated by OaBac5mini with the concentration of 50 μg/ml (2 × MICs) or 200 μg/ml (8 × MICs). The back arrows indicate the broken membrane.

The morphology and ultrastructure of *E. coli* ATCC 25922 treated with concentrations of 50 and 200 μg/ml OaBac5mini were investigated by SEM. As shown in Figure 7, *E. coli* treated with PBS (negative control) exhibited smooth and regular surfaces of the membranes. But both of the bacteria treated with OaBac5mini or 50 μg/ml PMB (positive control) exhibited atrophy, corrugation, and pore formation on the surface of the cell membranes, as well as the leakage of the intracellular contents. The results of SEM directly reflect the membrane damage, and also verify the results of NPN and DiSC_3_(5).

## Discussion

OaBac5 is a sheep-derived PrAMP consisting of a 6-residue N terminus followed by two copies of a 16-residue repeat and a 5-residue C terminus. Within the 16-residue repeat sequence is a combination of four repeats of the smaller 4-residue (XRRP), which is a shared characteristic in the amino acid sequences of Bac5 from different animals. Compared to that of bovine-derived Bac5, only five residues differences occur, which makes OaBac5 less hydrophobic and more cationic. Research demonstrated that OaBac5 showed potent activity against *E. coli, S. aureus*, and *C. albicans* (15). However, Bac5 only exhibited antibacterial activity against Gram-negative bacteria (27). Although OaBac5 is a potent broad-spectrum PrAMP, its complete amino acid sequence has not been obtained or synthesized *in vitro*. In 2004, OaBac5mini, a truncated fragment of OaBac5, was synthesized to contain the 6 N-terminal residues, one copy of the 16-residue repeat, and the first two residues of the second repeat. The susceptible tests against different strains showed that OaBac5mini had antibacterial activity similar to that of OaBac5, which implies that the full-length sequence is not required for the antibacterial activity of OaBac5 (17). In this study, we found that OaBac5mini showed stronger activity against four strains of MDR *E. coli* isolates (MICs, 1.95–3.26 μg/ml) than *E. coli* ATCC 25922 (MICs, 25 μg/ml). However, the MICs of OaBac5mini against *S. aureus* (MICs, 208.33 to >250 μg/ml) are far greater than those reported (MICs, 16–64 μg/ml) (17). We think that the main reasons are probably due to the different MIC test methods and strains used. Wu et al. performed the MICs experiment using a modified two-fold microtiter broth dilution method in which the antibacterial peptide was first diluted by a two-fold decrease in the buffer containing 0.2% bovine serum albumin and 0.01% acetic acid in Eppendorf tubes. At the same time, the LB medium used to culture the bacteria and test MICs did not contain NaCl (28). In this study, OaBac5mini was dissolved in purified water and bacteria were cultured in LB broth. It was reported that the antibacterial activity of OaBac5mini against *E. coli* O157:H7 increased with the decreasing concentrations of NaCl (18).

Salt ions in serum are important factors affecting the antibacterial activity of AMPs *in vivo* because they can bind to the negatively charged groups of lipopolysaccharide (LPS), a monolayer molecule on the outer membrane of Gram-negative bacteria (29). In this study, OaBac5mini exhibits similar antibacterial activity against *E. coli* ATCC 25922 in the presence of physiological concentrations of NaCl, KCl, NH_4_Cl, MgCl_2_ or FeCl_3_. But in the presence of physiological concentrations of CaCl_2_, the MIC of OaBac5mini increased four-fold and attained 100 μg/ml (Table 2). It has been reported that the presence of Ca^2+^ and Mg^2+^ caused the antibacterial activity of Bac5 against *E. coli* to significantly decrease. Moreover, Bac5 could kill *E. coli* more effectively by decreasing the concentration of NaCl (30). In addition, the antibacterial activity of OaBac5mini against *E. coli* O157:H7 was also affected by salt and metal ions. The presence of 100 mM NaCl significantly decreased the activity of OaBac5mini and its MIC attained about 30 μg/ml, which is similar to that of OaBac5mini against *E. coli* ATCC 25922 in the presence of 150 mM NaCl described in this study. Furthermore, the divalent ions Mg^2+^ and Ca^2+^ inactivated OaBac5mini at a concentration of 10 and 5 mM, respectively, which is higher than its corresponding physiological concentration (1 mM for Mg^2+^and 2.5 mM for Ca^2+^) in human serum (18). Taken together, OaBac5mini will keep antibacterial activity against *E. coli* in human serum. Apart from its stability in the presence of physiological concentrations of ions, we first investigated the effect of some gut factors on the antibacterial activity of OaBac5mini. The results showed that the activity of OaBac5mini against *E. coli* ATCC 25922 was not significantly affected by pH and pepsin, suggesting OaBac5mini may remain stable in the stomach. Furthermore, at concentrations of ≤ 10 μg/ml, proteinase K and trypsin also had no significant effect on the activity of OaBac5mini (Figure 1).

Physicochemical toxicity is an important limitation during the application of various antibacterial agents. In the present study, OaBac5mini showed no hemolysis, whose mean value of hemolysis ratio was −1.26 to 1.59% (Figure 3). In addition, all the concentrations of OaBac5mini rapidly promoted the proliferation of IPEC-J2 cells when it was added (Figure 4A) and IPEC-J2 cells showed increased cell indexes when treated with OaBac5mini (Figure 4B). The results demonstrate that OaBac5mini has no physicochemical toxicity, but can promote cell proliferation and survival. Cathelicidins are multifunctional peptides with diverse functions such as proliferation and migration of cells, immunoregulation, wound healing, angiogenesis and the release of cytokines (31). We previously found that PrAMP BSN-37 also promotes the proliferation of IPEC-J2 cells and Vero cells (13). Another cathelicidin derived from human LL-37 stimulated the proliferation of airway epithelial cells and wound closure (32). This may be because cathelicidins stimulate the expression of cytokines such as growth factors (33) and growth factor receptors (34), or activate the growth factor receptor-related signaling pathways (35).

The cell membrane is the first barrier of bacterial defense against external adverse factors. Most cathelicidin family AMPs contain amphiphilic α helix structures and are rich in positively charged amino acids. These kinds of AMPs accumulate on the surface of the negatively charged moieties in the cell wall through electrostatic attraction, such as LPS in the outer membranes of Gram-negative bacteria, and then disrupt the cytoplasmic membrane followed by cell lysis (36). However, some PrAMPs have been confirmed to kill Gram-negative bacteria mainly by binding with intracellular targets without apparent membrane damage (10, 37). Furthermore, the crystal structures of Bac7 (1–16), Bac7 (1–35) and Onc112 binding to the 70S ribosome from *Thermus thermophilus* showed that a common mechanism of action used by these PrAMPs is to inhibit protein synthesis (38, 39). In this study, OaBac5mini is a positively-charged hydrophilic linear peptide (Figure 5) with a net charge of 8, which is higher than the net charges of Bac5 and other truncated fragments (10). Furthermore, the significantly increased fluorescence intensity (Figure 6) and incomplete ultrastructure (Figure 7) indicated that OaBac5mini inhibited *E. coli* ATCC 25922 by damaging the cell membrane. However, when the transporter *sbmA* mainly responsible for the internalization of antibacterial peptides was inactivated in *E. coli* ATCC 25922, the *sbmA* deletion mutant showed a 4-fold increase of the MIC, suggesting OaBac5mini can enter into the cytoplasm and play its antibacterial activity. On the other hand, the time-kill curve showed that OaBac5mini could effectively kill *E. coli* ATCC 25922 at a concentration of 200 μg/ml. When the concentration of OaBac5mini is 50 or 100 μg/ml, the growth of bacteria was firstly inhibited and then, increased step by step (Figure 2). Therefore, the minimal inner membrane depolarization of bacteria treated by OaBac5mini with a low concentration was not enough to cause cell death. As a result, OaBac5mini plays its bacteriostatic role against *E. coli* by lytic and nonlytic modes of action. At present, some AMPs such as Bac7(1–35), Cathelicidin-BF, and Bac5(1–17) derivatives have been confirmed to kill Gram-negative bacteria by two different modes of action (40–42). Furthermore, Bac7(1–35) could not only bind to the ribosome to inhibit protein synthesis, but it can also bind to DnaK, which participates in purine metabolism and enriched kinase activity (43). It is evident that different action modes and multiple targets in the cytoplasm will make clinical strains unlikely to develop resistance against PrAMPs. Recently, Dolzani et al. (44) found that *Acinetobacter baumannii* AB5075 induced by sub-MICs of Bac7(1–35) for 16 passages did not produce resistance to Bac7(1–35). Taken together, PrAMPs are promising lead compounds for the development of the next antibiotics for treating clinical MDR bacterial infections.

## Conclusion

OaBac5mini is a potential and promising lead compound for the development of antibiotic substitutes with potent antibacterial activity against MDR *E. coli* isolates. Its activity was not seriously affected by *in vitro* condition factors (pH, temperature, repeated freeze-thawing) and *in vivo* condition factors (the serum salts, pepsin and proteinase K). Moreover, OaBac5mini was not hemolytic or cytotoxic. Furthermore, it kills *E. coli* by two different modes of action: inhibiting intracellular target(s) and damaging cell membranes. The next work is to search for the intracellular target(s) of OaBac5mini and illustrate its (their) antibacterial mechanism (s).

## Data availability statement

The original contributions presented in the study are included in the article/supplementary material, further inquiries can be directed to the corresponding author.

## Author contributions

Conceived and designed the experiments: YS, JHu, and JHe. Performed the experiments: SS, FR, and SF. Contributed reagents, materials, and analysis tools: SS and FR. Wrote the paper: SS, YS, JB, and PS. All authors contributed to the article and approved the submitted version.
